# Estimated Phytate Intake Is Associated with Improved Cognitive Function in the Elderly, NHANES 2013–2014

**DOI:** 10.3390/antiox10071104

**Published:** 2021-07-10

**Authors:** Doreen Y Larvie, Seth M Armah

**Affiliations:** Department of Nutrition, University of North Carolina at Greensboro, Greensboro, NC 27412, USA; dylarvie@uncg.edu

**Keywords:** phytate, cognitive decline, aging, elderly, Digit Symbol Substitution test

## Abstract

Phytate, an antioxidant, may improve cognition by inhibiting iron catalyzed hydroxyl radical formation. Particularly in the elderly, this provides a potential dietary approach for mitigating age-related brain neuronal dysfunction and loss. In this study, we investigated the relationship between phytate intake and cognitive function in the elderly. We used data from the 2013–2014 National Health and Nutrition Examination Survey (NHANES) and the corresponding Food Patterns Equivalents Database (FPED). Phytate content of food groups from published data were merged with the appropriate FPED data to estimate the total phytate intake for each subject. Principal component analysis was used to develop a composite score from four cognitive function scores in NHANES data, and regression analysis was used to determine the relationship between this score and phytate intake. Median phytate intake was 0.65 (0.61, 0.71) g/day. It was low among females, non-Hispanic blacks, and people with history of at least one chronic disease (*p* < 0.05). In regression analysis adjusted for confounders, phytate intake was positively associated with cognitive function (β (95% CI) = 1.90 (0.73–3.07); *p* = 0.015). These results suggest that phytate may be associated with improved cognition, hence the need to consider including phytate-rich foods in the diet among the elderly.

## 1. Introduction

Cognition encompasses a spectrum of higher order cerebral function from normal to subjective complaints to evidence of decline in cerebral function to dementia [1]. Older age is a known risk factor for dementia, which affects approximately 10 million people worldwide yearly with 150 million people estimated to be living with the condition by 2050 [2,3]. Aging and age-related disorders accelerate brain neuronal dysfunction and loss resulting in decline in processing speed, attention and executive function [1,4]. 

Cognitive decline in aging is multifaceted [4] and is attributed to factors such as impaired calcium homeostasis, mitochondrial dysfunction, oxidative damage and inflammation and increased susceptibility to stress leading to epigenetic modifications that affect learning, memory and synaptic processes in the brain [5]. Inadequate intake of certain food groups including fruits, vegetables, cereals, and grains, as well as nutrients such as zinc, selenium, copper, fiber, and some vitamins have also been linked with cognitive decline [6,7]. In addition to nutrients and food groups, phytochemicals such as phytates and some polyphenols are potential agents for improving cognitive health in aging due to their anti-oxidant and anti-inflammatory properties [8]. 

Phytate (myo-inositol hexakisphosphate), a salt of phytic acid with six phosphate groups and an inositol ring is a reservoir for phosphorus needed for germination of plants and seeds [8]. It is known that the ability of phytate to chelate iron, zinc, copper and magnesium may decrease the bioavailability and absorption of these minerals from the diet [9]. However, this chelation property is dependent on the ratio of phytate to metal. Hence, consuming a healthy and balanced diet with adequate proportions of these trace metals can minimize this chelation property and not result in mineral deficiency [10,11]. As an antioxidant, phytic acid accelerates the oxidation of Fe^2+^ to Fe^3+^, thus minimizing availability of Fe^2+^ for iron catalyzed hydroxyl radical formation via the Fenton reaction [11,12,13]. In this way, phytate may prevent lipid peroxidation, and hence, acts to mitigate inflammation and neurodegenerative diseases [10,12,14,15]. In mice models of Alzheimer’s disease—the commonest age-related neurodegenerative disease—a 2% phytic acid diet, resulted in an increase in cytochrome oxidase as a measure of mitochondrial function and a decrease in malondialdehyde (MDA) in the treatment group compared to wild type controls. MDA is a by-product of oxidative stress, lipid peroxidation and cell membrane damage [16]. Increased MDA may be observed in brain regions of Alzheimer’s disease patients and in aged rats showing cognitive deficits [17,18]. In MC65 human neuroblastoma cells, 100 µM phytic acid in the presence of tetracycline resulted in reduced concentrations of hydrogen peroxide and increased concentrations of proteins responsible for autophagy and housekeeping of the neuroblastoma cells (beclin-1 and SIRT1) [12]. These findings are supported by results from a Parkinson’s disease rat cell line where phytic acid also decreased 6-hydroxydopamine-induced apoptosis and DNA fragmentation compared to non-treated cells in both normal and iron excess conditions [19].

In spite of the protective effect of phytic acid seen in rodent and cell studies, no human study has investigated the relationship between phytate consumption and cognitive function, especially in the elderly who are vulnerable to age-related cognitive decline. The aim of this study was to investigate this relationship using data from the NHANES 2013–2014 cycle. We hypothesized that phytate intake will be associated with improved cognitive function among the elderly.

## 2. Materials and Methods

### 2.1. Data Source and Study Population

Data for older adults 60 years or more from the 2013–2014 National Health and Nutrition Examination Survey (NHANES) and the corresponding Food Patterns Equivalents Database (FPED version 2013–2014) were used for this study. NHANES is a complex multi-stage survey collected in 2-year cycles to assess the health and nutritional status of non-institutionalized children and adults in the US [20]. Data collected include interviews for demographic and dietary data, laboratory tests and physical examination. FPED is used to evaluate whether the food and beverage intake of Americans meets the recommendations of the dietary guidelines for Americans. It converts food and beverages from What We Eat in America (WWEIA), NHANES into 37 food pattern components. Since phytate intake data is not available from the NHANES data, the estimated phytate content of different food groups (dark green leafy vegetables, potatoes, other starchy vegetables, other vegetables, legumes, whole grains, refined grains, soy products, and nuts and seeds) were obtained from the Food and Agriculture Organization/International Network of Food Data Systems/International Zinc Nutrition Consultative Group (FAO/INFOODS/IZiNCG) database [21]. This data was merged with the appropriate FPED data for the 2013–2014 survey cycle of WWEIA to estimate phytate intake of the NHANES participants [22]. Informed consent was obtained from all individual participants and all procedures performed involving human subjects in NHANES were in accordance with the Declaration of Helsinki (IRB number 2011–17).

### 2.2. Cognitive Assessments

Cognitive functioning is measured periodically in NHANES as part of the household survey or in the Mobile Examination Center among individuals aged 60 years or more. Assessments include (1) word learning and recall modules based on the Consortium to Establish Registry for Alzheimer’s disease (CERAD), (2) Animal Fluency, and (3) the Digit Symbol Substitution test (DSST). The CERAD Word List Learning and Recall tests are used to ascertain immediate and delayed learning of new verbal information. In the CERAD Word List Learning, participants are required to read aloud 10 words in three trials with the order of the words in each trial altered. In both the word learning and recall modules, the highest points are equal to 10. The CERAD Recall testing occurs after the Animal Fluency and Digit Symbol Substitution tests. The Animal Fluency test is used to measure categorical verbal fluency; participants are asked to name animals in a 1-min duration and the scores obtained equal the number of animals named correctly. The DSST is obtained from the Weschler Intelligence Scale and is used to assess processing speed, working memory and sustained attention. This is a paper-based test with a key at the top containing nine numbers and their corresponding symbols. Participants have 2 min to draw these corresponding symbols into 133 boxes containing their respective numbers. The scores represent the number of symbols correctly drawn. In all cognitive function tests, higher scores indicate higher cognitive function [23,24].

### 2.3. Covariates

A variety of covariates known to be related to cognitive function and phytate intake were included in the analyses. These were race/ethnicity (Mexican-American, Non-Hispanic White, Non-Hispanic Black and Non-Hispanic Asian), tobacco smoking, alcohol consumption, poverty to income ratio, marital status, education, and medical condition history [23]. Smoking was categorized as follows: (1) individuals who reported smoking at least 100 cigarettes in their lifetime and smoke every day or some days were classified as current smokers; (2) those who have smoked at least 100 cigarettes in their lifetime and now do not smoke were classified as former smokers; (3) those who have not smoked 100 cigarettes in their lifetime were classified as never smokers. Alcohol consumption was classified as: (1) moderate drinkers (<8 drinks/week); (2) heavy drinkers (8 drinks or more/week). Ratio of family income to poverty guidelines was classified as: (1) low family income to poverty ratio (≤0.99); (2) high family income to poverty ratio (≥1.00). Marital status was classified as: (1) married/living with partner; (2) widowed/divorced/separated; (3) never married. Educational status was classified as: (1) less than high school; (2) high school; (3) college educated. Subjects were considered to have a medical condition history if they reported at least one of these conditions, stroke, diabetes, coronary heart disease, coronary heart failure, heart attack, hypertension. Otherwise, they were considered to have no medical condition history. Age group was classified as: (1) old adults from 60 to 70 years; (2) older adults from 71 to 80 years.

### 2.4. Statistical Analyses

Data were analyzed using R Studio version 1.2.5001 (R Core Team, Vienna, Australia). Analysis was performed using the “survey” package accounting for sampling weight, strata, and primary sampling unit in the survey design. The four cognitive scores (CERAD word learning and recall scores, animal fluency, and DSST) were used in principal component analysis to generate the different principal components. Descriptive statistics used for the study population were median (95% confidence intervals) and percentages. Mood’s median test was used to compare medians (95% CI) between groups and Benjamini–Hochberg correction was used to adjust for false discovery rate. Stepwise multiple linear regression with backward elimination of non-significant variables was used to determine the association between cognitive function and phytate intake. We used the first principal component scores (containing largest variance) and DSST as measures of cognitive function in the regression models. This is because the DSST score is a good measure for evaluating frontal lobe-related functions including visuospatial skills, sustained attention, and motor speed-of-processing [25,26]. In both models the following covariates were adjusted for: age, sex, daily fiber intake, educational status, medical condition history, and poverty to income ratio. Since phytate and fiber are mostly from the same foods and some studies have reported an association between fiber intake and cognitive function, we kept fiber in all regression models even though it was not significant. Statistical significance was set at *p* ≤ 0.05. 

## 3. Results

In Table 1, data for phytate intake was available for 1567 of study participants. While Non-Hispanic Whites (77%) formed a majority of the sample population, Non-Hispanic Asians (4%) and Mexican-American (4%) were the smallest ethnic groups. Half (50%) of the study participants had never smoked, and a majority (70%) had a history of at least one medical condition. 

**Figure 1:**
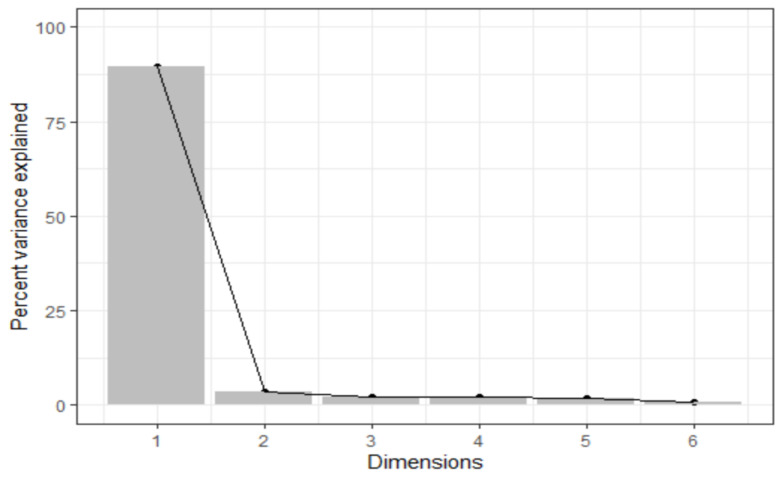
Scree plot showing the variance contributed by the principal components for the cognitive function tests.

The results of the principal component analysis are shown in Figure 1. The first principal component score loading contributed a majority (90%) of the variance in the loadings. 

From Table 2, the daily median phytate intake was significantly higher for males (0.74 (0.68, 0.79) g/day) than females (0.6 (0.54, 0.65) g/day, *p* = 0.0002). Similarly, fiber intake was higher in males (17 (15.8, 18.3) g/day) compared to females (13.7 (13, 14.5) g/day, *p* < 0.0001). Non-Hispanic Whites (0.67 (0.62, 0.73) g/day), Non-Hispanic Asians (0.8 (0.67, 0.91) g/day) and Mexican-American (0.69 (0.59, 0.84) g/day) all consumed significantly more phytate than their Non-Hispanic Black counterparts (0.5 (0.45, 0.59) g/day). Phytate intake was significantly higher among those who had never smoked (0.67 (0.61, 0.75) g/day) and former smokers (0.66 (0.61, 0.73) g/day) compared to current smokers (0.5 (0.45, 0.6) g/day). DSST scores for females (54 (53, 56)) were significantly higher than for males (51 (49, 53); *p* = 0.0001). Compared to those with no medical condition history, DSST scores for study participants with at least one medical condition history were significantly lower (57 (54, 59) versus 51 (49, 53); *p* = 0.0003). The trend in DSST scores for medical condition history were confirmed with the first principal component scores as those with at least one medical condition had lower scores (1.18 (0.89, 1.41)) compared to those without a medical condition history (1.7 (1.38, 1.99), *p* = 0.0004). Also, individuals who had never smoked and former smokers had significantly higher DSST scores compared to current smokers. When regression analysis was used to determine the association between phytate intake and cognitive function adjusting for potential confounders, phytate intake showed a significant positive association with both the first principal component score (1.9 (0.73, 3.07); *p* = 0.015) and DSST (0.23 (0.13, 0.33); *p* = 0.003, Table 3). This finding was independent of sex, age group, fiber intake, medical condition history, income to poverty ratio and education status. 

## 4. Discussion and Conclusions

In this study, among adults 60 years and older, daily phytate intake was positively associated with cognitive function after controlling for potential confounders. Phytate is a naturally occurring phosphorus compound found in high fiber food sources with global estimates for daily intake ranging from 0.18 to 4.57 g/day, depending on type of diet and preparation methods used [10,27,28]. For example, the median phytate intake for the UK is 0.81 g/day, while higher intake is reported in countries with a predominantly plant-based diet as seen in Nigeria (2.2 g/day) [29,30] and South Korea (1.68 g/day) [31]. The median phytate intake in this study was 0.65 g/day, similar to 0.6 g/day reported among adults 20 years and older [22].

Aging is associated with cognitive decline due to accelerated brain neuronal dysfunction and loss resulting in decline in processing speed, attention and executive function [1,4]. Among adults, neurodegenerative diseases such as Parkinson’s Disease and Alzheimer’s Disease result in motor function abnormalities, dementia, sleep disturbances, memory problems, and death [32]. Studies also show that age-related conditions such as stroke, diabetes, hypertension and tobacco smoking may be associated with cognitive impairment [33] and as seen in our study, having at least one of these medical conditions was associated with cognitive decline measured by DSST scores. Studies show that low physical activity, a reduced involvement in activities of daily living, an increased risk of cardiovascular disease, high blood pressure and poor glycemic control among older adults may hasten cognitive decline [34,35]. Additionally, the contribution of some dietary patterns in slowing cognitive decline in the elderly has been studied. Among adults over 65 years with a previous history of stroke, being in the highest tertile of a Mediterranean and DASH diet score was associated with a slower rate of cognitive decline compared to those in the lowest tertile [36]. Phytochemicals such as phytates are potential agents for improving cognitive health in aging, as suggested by the findings of our study, due to their antioxidant and anti-inflammatory properties [7].

Phytic acid is an antioxidant and may prevent iron-related free radical generation to protect against neurodegeneration, hence mitigating neuronal damage and loss [19,37]. Brain tissue is particularly susceptible to oxidative stress due to the high levels of polyunsaturated fatty acids, low antioxidant concentrations (superoxide dismutase and catalase lower than in liver tissue) and the high oxidative stress environment [19]. Grases et al. [38] have shown that phytic acid can cross the blood–brain barrier by demonstrating a ten-fold increase in rat brain concentrations of phytic acid compared to other tissues after a 10 g phytic acid purified diet. 

While there is a dearth of data showing the association between phytate intake and cognitive function in the elderly, our study showed that phytate intake was positively associated with cognitive function among adults 60 years and older. This finding concurs with a study among infants six to sixty months old where the mean phytate intake from complementary foods was associated with higher scoring trajectories in cognitive function based on the Bayley-III scale and the Wechsler Preschool and Primary Scale of Intelligence [39]. 

The role of phytate in cognition has also been reported in several animal studies. In a female mouse model of Alzheimer’s disease, phytic acid improved mitochondrial function and reduced lipid peroxidation markers [12]. Similarly, in a rat model of Parkinson’s disease fed 100 mg/kg phytic acid, there was a reduction in apomorphine-induced rotations, which are indicators of nigrostriatal dopamine depletion [37]. These findings are also supported in Parkinson’s disease rat immortalized mesencephalic dopaminergic neuronal cell lines where treatment with phytic acid decreased 6-hydroxydopamine-induced apoptosis by reducing caspase-3 activity and DNA fragmentation in both normal and iron excess conditions [19]. On the other hand, our study, showed that females had higher DSST scores—in spite of the lower phytate intake—than their male counterparts., suggesting that sex differences may be evident in the relationship between phytate and cognition. 

The strengths of this study includes the large sample size, the use of a majority of covariates known to influence cognitive function and data reduction of cognitive function scores using principal component analysis to ensure the variability of each of the cognitive function scores were well accounted for. However, the use of principal component scores also limits the explanation of the effect sizes observed. Additionally, since we estimated phytate intake from foods consumed, other bioactive components of the foods beyond fiber such as polyphenols may contribute to the positive association between phytate intake and cognition observed in this study. Future studies using pure phytic acid are warranted to determine its independent role in cognitive function. Another limitation of our study is its cross-sectional nature, which does not support causal inference. 

In conclusion, we have demonstrated a positive association between phytate intake and cognitive function among adults 60 years or older in this study. Future longitudinal studies as well as mechanistic studies are recommended to further understand the relationship between phytate intake and cognition in the elderly. 

## Figures and Tables

**Table 1:** Background characteristics of older adults 60 years and above, NHANES 2013–2014.

Demographic Data	*n* (Unadjusted)	Percentage (Adjusted) ^1^
Total	1841	100
Sex		
Male	967	54
Female	874	46
Ethnicity ^2^		
Non-Hispanic White	896	77
Non-Hispanic Black	388	9
Non-Hispanic Asian	168	4
Mexican-American	210	4
Smoking status		
Never	917	50
Current user	232	10
Former user	690	40
Medical history ^3^		
None	507	30
At least one	1334	70

^1^ Adjusted for sampling weight, strata, and primary sampling unit; ^2^ estimates of “other” race not reported but included in analysis; ^3^ medical history includes stroke, coronary heart disease, coronary heart failure, diabetes, heart attack and hypertension.

**Table 2:** Phytate intake, fiber intake, cognitive function and principal component scores (from cognitive function) of adults 60 years and above, NHANES 2013–2014 ^1^.

Demographic Data	Phytate Intake (g/day)	Fiber Intake (g/day)	Digit Symbol Substitution Score (DSST)	Animal Fluency Score	CERAD Recall Score	CERAD Learning Score	Principal Component Score
Total	0.65 (0.61, 0.71)	15 (14.4, 15.8)	53 (51, 54)	18 (17, 18)	7 (6,7)	7 (6.7, 7)	1.35 (1.07, 1.66)
Sex							
Women	0.6 (0.54, 0.65) ^a^	13.7 (13, 14.5) ^a^	54 (53, 56) ^a^	18 (17, 18) ^a^	7 (7, 7) ^a^	7.3 (7, 7.3) ^a^	1.35 (1.07, 1.62) ^a^
Men	0.74 (0.68, 0.79) ^b^	17 (15.8, 18.3) ^b^	51 (49, 53) ^b^	18 (17, 19) ^a^	6 (6, 7) ^b^	6.7 (6.3,7) ^b^	1.33 (0.97, 1.7) ^a^
Ethnicity ^2^							
Non-Hispanic White	0.67 (0.62, 0.73) ^a^	15 (14.4, 16) ^a^	54 (53, 56) ^a^	18 (18, 19) ^a,b^	7 (7, 7) ^b^	7 (7, 7.3) ^b^	1.85 (1.68, 2) ^a^
Non-Hispanic Black	0.5 (0.45, 0.59) ^b^	13 (12, 14.1) ^b^	40 (36, 44) ^b^	14 (13, 15) ^b^	6 (6, 7) ^a^	6.7 (6.3, 7) ^a^	−1.2 (−1.62, −0.87) ^b^
Non-Hispanic Asian	0.8 (0.67, 0.91) ^a^	17.2 (15.2, 18.8) ^a^	53 (52, 55) ^a,b^	14 (13, 15) ^b^	7 (7, 8) ^b^	7 (6.7, 7) ^b^	−0.31 (−0.64, −0.11) ^a,b^
Mexican-American	0.69 (0.59, 0.84) ^a^	17.6 (14.5, 20.4) ^a,b^	41 (37, 45) ^b^	17 (16, 17) ^a^	6 (5, 7) ^a^	6.3 (5.7, 6.7) ^a,b^	−1.5 (−1.96, −1.18) ^b^
Smoking status							
Never	0.67 (0.61, 0.75) ^a^	15.6 (14.7, 16.1) ^a^	54 (52, 56) ^a^	18 (17, 19) ^a^	7 (7, 7) ^a^	7 (6.7, 7.3) ^a^	1.33 (1.03, 1.68) ^a^
Current user	0.5 (0.45, 0.6) ^b^	10.8 (9.4, 12.3) ^b^	49 (44, 53) ^b^	16 (15, 18) ^b^	7 (6, 7) ^b^	7 (6.7, 7) ^b^	0.79 (0.25, 1.53) ^b^
Former user	0.66 (0.61, 0.73) ^a^	15.6 (14.7, 16.7) ^a^	51 (48, 54) ^a^	18 (17, 19) ^a^	7 (6, 7) ^b^	7 (6.7, 7) ^b^	1.48 (1.07, 1.81) ^a^
Medical history ^3^							
None	0.75 (0.71, 0.8) ^a^	16.3 (15, 17.4) ^a^	57 (54, 59) ^a^	19 (18, 20) ^a^	7 (7, 7) ^a^	7.3 (7, 7.3) ^a^	1.7 (1.38, 1.99) ^a^
At least one	0.62 (0.58, 0.65) ^b^	14.4 (13.3, 15.2) ^b^	51 (49, 53) ^b^	17 (17, 18) ^b^	7 (6, 7) ^b^	6.7 (6.7, 7) ^b^	1.18 (0.89, 1.41) ^b^

Values with dissimilar superscripts differ significantly (*p* ≤ 0.05, Benjamini–Hochberg correction for multiple comparisons), ^1^ Values are median (95% CI), ^2^ estimates of “other” race not reported but included in analysis; ^3^ medical history includes stroke, coronary heart disease, coronary heart failure, diabetes, heart attack and hypertension. Values with dissimilar superscripts differ significantly (*p* ≤ 0.05, Benjamini–Hochberg correction for multiple comparisons).

**Table 3:** Association between phytate intake and cognitive function (Digit Symbol score and first principal component score) among older adults 60–80 years old ^1^.

Demographic/Nutrient Intake	Digit Symbol Substitution Score			Principal Component Score		
Predictors	β	CI	*p*	β	CI	*p*
(Intercept)	35.33	27.14, 43.52	<0.001	−1.12	−1.66, −0.58	0.005
Phytate intake (g/day) ^2^	1.90	0.73, 3.07	0.015	0.23	0.13, 0.33	0.003
Sex						
Male	RG					
Female	5.16	3.48, 6.83	0.001	0.26	0.12, 0.40	0.009
Fiber intake (g/day) ^2^	1.10	−0.51, 2.71	0.222	−0.04	−0.21, 0.13	0.675
Age group						
Old adult (60–70 years)	RG					
Older adult (71–80 years)	−9.83	−11.40, −8.26	<0.001	−0.77	−0.92, −0.62	<0.001
Medical condition history ^3^						
None	RG					
At least one	−2.74	−4.52, −0.96	0.020	−0.21	−0.35, −0.06	0.025
Income to poverty ratio						
≤0.99	RG					
≥1.00	8.44	6.02, 10.87	<0.001	1.24	1.01, 1.48	<0.001
Educational status						
College educated	RG					
High school	−8.02	−9.99, −6.05	<0.001	−0.63	−0.82, −0.45	<0.001
Less than high school	−22.08	−26.71, −17.45	<0.001	−1.67	−2.32, −1.03	0.001
Observations	1353			1340		
R^2^	0.322			0.277		

RG, reference group; ^1^ *p*-values are based on multiple regression analysis; ^2^ Values were log-transformed before analysis; ^3^ Medical history includes stroke, coronary heart disease, coronary heart failure, diabetes, heart attack and hypertension.

## Data Availability

Publicly available datasets were analyzed in this study. This data can be found here: (https://wwwn.cdc.gov/nchs/nhanes/continuousnhanes/overview.aspx?BeginYear=2013 accessed on 11 April 2019).

## References

[1] Cohen J.A., Verghese J., Zwerling J.L. (2016). Cognition and gait in older people. Maturitas.

[2] Prince M., Bryce R., Albanese E., Wimo A., Ribeiro W., Ferri C. (2013). The global prevalence of dementia: A systematic review and metaanalysis. Alzheimer Dement..

[3] Dementia. https://www.who.int/news-room/fact-sheets/detail/dementia.

[4] Murman D.L. (2015). The Impact of Age on Cognition. Semin. Hear..

[5] Harman M.F., Martín M.G. (2020). Epigenetic mechanisms related to cognitive decline during aging. J. Neurosci. Res..

[6] Bruins M.J., Van Dael P., Eggersdorfer M. (2019). The Role of Nutrients in Reducing the Risk for Noncommunicable Diseases during Aging. Nutrients.

[7] Li S., Sun W., Zhang D. (2019). Association of Zinc, Iron, Copper, and Selenium Intakes with Low Cognitive Performance in Older Adults: A Cross-Sectional Study from National Health and Nutrition Examination Survey (NHANES). J. Alzheimers Dis..

[8] Raboy V. (2003). myo-Inositol-1,2,3,4,5,6-hexakisphosphate. Phytochemistry.

[9] Fardet A. (2010). New hypotheses for the health-protective mechanisms of whole-grain cereals: What is beyond fibre?. Nutr. Res. Rev..

[10] Reddy N.R., Sathe S.K. (2001). Food Phytates.

[11] Graf E., Empson K.L., Eaton J.W. (1987). Phytic acid. A natural antioxidant. J. Biol. Chem..

[12] Anekonda T.S., Wadsworth T.L., Sabin R., Frahler K., Harris C., Petriko B., Ralle M., Woltjer R., Quinn J.F. (2011). Phytic Acid as a Potential Treatment for Alzheimer’s Pathology: Evidence from Animal and in vitro Models. J. Alzheimers Dis..

[13] Silva E.O., Bracarense A.P.F.R.L. (2016). Phytic Acid: From Antinutritional to Multiple Protection Factor of Organic Systems. J. Food Sci..

[14] Fulcher R., O’Brien P.T., Wong I.S. (1981). Microchemical detection of niacin, aromatic amine, and phytin reserves in cereal bran. Cereal. Chem..

[15] O’Dell B.L., De Boland A.R., Koirtyohann S.R. (1972). Distribution of phytate and nutritionally important elements among the morphological components of cereal grains. J. Agric. Food Chem..

[16] Talarowska M., Gałecki P., Maes M., Gardner A., Chamielec M., Orzechowska A., Bobińska K., Kowalczyk E. (2011). Malondialdehyde plasma concentration correlates with declarative and working memory in patients with recurrent depressive disorder. Mol. Biol. Rep..

[17] Zabel M., Nackenoff A., Kirsch W.M., Harrison F.E., Perry G., Schrag M. (2018). Markers of oxidative damage to lipids, nucleic acids and proteins and antioxidant enzymes activities in Alzheimer’s disease brain: A meta-analysis in human pathological specimens. Free Radic. Biol. Med..

[18] González-Fraguela M.E., Blanco-Lezcano L., Fernandez-Verdecia C.I., Serrano Sanchez T., Robinson Agramonte M.D.L.A., Cardellá Rosales L.L. (2018). Cellular Redox Imbalance and Neurochemical Effect in Cognitive-Deficient Old Rats. Behav. Sci..

[19] Xu Q., Kanthasamy A.G., Reddy M.B. (2011). Phytic Acid Protects against 6-Hydroxydopamine-Induced Dopaminergic Neuron Apoptosis in Normal and Iron Excess Conditions in a Cell Culture Model. https://www.hindawi.com/journals/pd/2011/431068/abs/.

[20] NHANES 2013–2014 Overview. https://wwwn.cdc.gov/nchs/nhanes/ContinuousNhanes/Overview.aspx?BeginYear=2013.

[21] Brown K.H., Rivera J.A., Bhutta Z., Gibson R.S., King J.C., Lönnerdal B., Ruel M.T., Sandtröm B., Wasantwisut E., International Zinc Nutrition Consultative Group (IZiNCG) (2004). International Zinc Nutrition Consultative Group (IZiNCG) technical document #Assessment of the risk of zinc deficiency in populations and options for its control. Food Nutr. Bull..

[22] Armah S.M. (2019). Association between Phytate Intake and C-Reactive Protein Concentration among People with Overweight or Obesity: A Cross-Sectional Study Using NHANES 2009/2010. Int. J. Environ. Res. Public Health.

[23] Li H., Wang Z., Fu Z., Yan M., Wu N., Wu H., Yin P. (2018). Associations between blood cadmium levels and cognitive function in a cross-sectional study of US adults aged 60 years or older. BMJ Open.

[24] NHANES 2013–2014: Cognitive Functioning Data Documentation, Codebook, and Frequencies. https://wwwn.cdc.gov/Nchs/Nhanes/2013-2014/CFQ_H.htm.

[25] Rosano C., Simonsick E.M., Harris T.B., Kritchevsky S.B., Brach J., Visser M., Yaffe K., Newman A.B. (2004). Association between Physical and Cognitive Function in Healthy Elderly: The Health, Aging and Body Composition Study. Neuroepidemiology.

[26] Tsai C.-K., Kao T.-W., Lee J.-T., Wu C.-J., Hueng D.-Y., Liang C.-S., Wang G.-C., Yang F.-C., Chen W.-L. (2016). Increased risk of cognitive impairment in patients with components of metabolic syndrome. Medicine.

[27] Schlemmer U., Frølich W., Prieto R.M., Grases F. (2009). Phytate in foods and significance for humans: Food sources, intake, processing, bioavailability, protective role and analysis. Mol. Nutr. Food Res..

[28] Buades Fuster J.M., Sanchís Cortés P., Perelló Bestard J., Grases Freixedas F. (2017). Plant phosphates, phytate and pathological calcifications in chronic kidney disease. Nefrol. Engl. Ed..

[29] Amirabdollahian F., Ash R. (2010). An estimate of phytate intake and molar ratio of phytate to zinc in the diet of the people in the United Kingdom. Public Health Nutr..

[30] Harland B.F., Peterson M. (1978). Nutritional status of lacto-ovo vegetarian Trappist monks. J. Am. Diet. Assoc..

[31] Kwun I.-S., Kwon C.-S. (2000). Dietary Molar Ratios of Phytate: Zinc and Millimolar Ratios of Phytate × Calcium:Zinc in South Koreans. Biol. Trace Elem. Res..

[32] Obisesan T., Gillum R.F. (2009). Cognitive function, social integration and mortality in a U.S. national cohort study of older adults. BMC Geriatr..

[33] Lo Coco D., Lopez G., Corrao S. (2016). Cognitive impairment and stroke in elderly patients. Vasc. Health Risk Manag..

[34] Landau S.M., Marks S.M., Mormino E.C., Rabinovici G.D., Oh H., O’Neil J.P., Wilson R.S., Jagust W.J. (2012). Association of Lifetime Cognitive Engagement and Low β-Amyloid Deposition. Arch Neurol Am. Med Assoc..

[35] Rovner B.W., Casten R.J., Leiby B.E. (2016). Determinants of Activity Levels in African Americans With Mild Cognitive Impairment. Alzheimer Dis. Assoc. Disord..

[36] Cherian L., Wang Y., Fakuda K., Leurgans S., Aggarwal N., Morris M. (2019). Mediterranean-Dash Intervention for Neurodegenerative Delay (MIND) Diet Slows Cognitive Decline After Stroke. J. Prev. Alzheimers Dis..

[37] Rahmati B., Khalili M., Hamoleh-Shalali Z., Roghani M., Baluchnejadmojarad T. (2015). Phytic Acid Mitigates Motor Asymmetry in Male Rat with Unilateral 6-Hydroxydopamine Striatal Lesion. J. Basic Clin. Pathophysiol..

[38] Grases F., Simonet B.M., Prieto R.M., March J. (2001). Phytate levels in diverse rat tissues: Influence of dietary phytate. Br. J. Nutr..

[39] McCormick B., Caulfield L., Richard S., Pendergast L., Murray-Kolb L. (2019). MAL-ED Network Investigators Nurturing Environments and Nutrient-Rich Diets May Improve Cognitive Development: Analysis of Cognitive Trajectories from Six to Sixty Months from the MAL-ED Study (OR10-01-19). Curr. Dev. Nutr..

